# Bis(1-phenyl-3-{(*Z*)-[phen­yl(pyridin-2-yl)methyl­idene]amino-κ^2^ *N*,*N*′}urea-κ*O*)nickel(II) dinitrate

**DOI:** 10.1107/S1600536812026207

**Published:** 2012-06-16

**Authors:** N. Aiswarya, Jinsa Mary Jacob, M. R. Prathapachandra Kurup, Seik Weng Ng

**Affiliations:** a Department of Applied Chemistry, Cochin University of Science and Technology, Kochi 682 022, India; b Department of Chemistry, University of Malaya, 50603 Kuala Lumpur, Malaysia; c Chemistry Department, King Abdulaziz University, PO Box 80203 Jeddah, Saudi Arabia

## Abstract

The Ni^II^ atom in the title salt, [Ni(C_19_H_16_N_4_O)_2_](NO_3_)_2_, is *N*,*N*′,*O*-chelated by two neutral Schiff base ligands in a distorted octa­hedral geometry. One nitrate ion inter­acts with the metal atom indirectly, in an outer-sphere type of coordination, through N—H⋯O hydrogen bonds; the other nitrate ion does not engage in any inter­actions and is equally disordered over two positions in the crystal.

## Related literature

For related copper(II) adducts, see: Patel (2010 ▶); Patel *et al.* (2009 ▶, 2010 ▶).
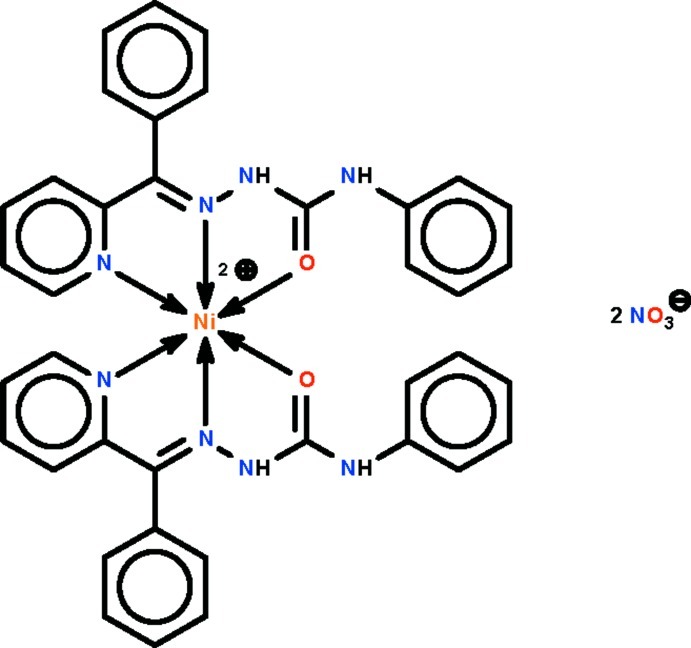


## Experimental

### 

#### Crystal data

[Ni(C_19_H_16_N_4_O)_2_](NO_3_)_2_
*M* *_r_* = 815.45
Monoclinic, 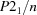

*a* = 14.0112 (3) Å
*b* = 16.0445 (3) Å
*c* = 16.4939 (3) Å
β = 95.959 (1)°
*V* = 3687.84 (12) Å^3^
*Z* = 4
Mo *K*α radiation
μ = 0.60 mm^−1^
*T* = 293 K
0.35 × 0.30 × 0.20 mm

#### Data collection

Bruker APEXII diffractometer
Absorption correction: multi-scan (*SADABS*; Sheldrick, 1996 ▶) *T*_min_ = 0.819, *T*_max_ = 0.890
51193 measured reflections
6527 independent reflections
4632 reflections with *I* > 2σ(*I*)
*R*_int_ = 0.070

#### Refinement

*R*[*F* ^2^ > 2σ(*F* ^2^)] = 0.053
*wR*(*F* ^2^) = 0.169
*S* = 1.09
6527 reflections
544 parameters
62 restraints
H-atom parameters constrained
Δρ_max_ = 1.11 e Å^−3^
Δρ_min_ = −0.52 e Å^−3^

### 

Data collection: *APEX2* (Bruker, 2004 ▶); cell refinement: *SAINT* (Bruker, 2004 ▶); data reduction: *SAINT*; program(s) used to solve structure: *SHELXS97* (Sheldrick, 2008 ▶); program(s) used to refine structure: *SHELXL97* (Sheldrick, 2008 ▶); molecular graphics: *X-SEED* (Barbour, 2001 ▶); software used to prepare material for publication: *publCIF* (Westrip, 2010 ▶).

## Supplementary Material

Crystal structure: contains datablock(s) global, I. DOI: 10.1107/S1600536812026207/xu5561sup1.cif

Structure factors: contains datablock(s) I. DOI: 10.1107/S1600536812026207/xu5561Isup2.hkl

Additional supplementary materials:  crystallographic information; 3D view; checkCIF report

## Figures and Tables

**Table 1:** Hydrogen-bond geometry (Å, °)

*D*—H⋯*A*	*D*—H	H⋯*A*	*D*⋯*A*	*D*—H⋯*A*
N3—H3⋯O3	0.88	2.14	2.983 (4)	162
N4—H4⋯O4	0.88	2.11	2.869 (5)	145
N7—H7⋯O3^i^	0.88	2.11	2.955 (4)	162
N8—H8⋯O5^i^	0.88	2.01	2.819 (4)	152

Symmetry code: (i) 
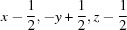
.

**Fig. 1.**
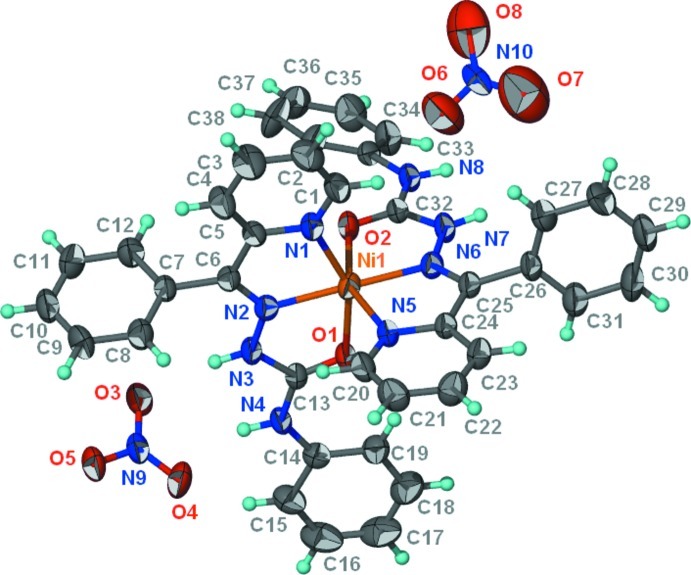
Thermal ellipsoid plot (Barbour, 2001) of [Ni(C19H16N4O)2] 2NO3 at the 50% probability level; hydrogen atoms are drawn as spheres of arbitrary radius. The disorder in one of the nitrate ions is not shown.

## supplementary crystallographic information

### Comment

Of the plethora of transition metal derivatives of Schiff bases whose crystal structures have been reported, the adducts of 2-benzoylpyridine semicarbazone are limited to those of copper(II) only. In these (Patel, 2010; Patel *et al.*, 2009; Patel *et al.*, 2010), the neutral ligand *N*,*N*',*O*-chelates to the metal atom. In the nickel(II) nitrate derivative (Scheme I), the Ni atom is *N*,*N*',*O*-chelated by two neutal Schiff base ligands in an octahedral geometry. One nitrate interacts with the metal atom indirectly, in an outer-sphere type of coordination, through N–H···O hydrogen bonds. The other nitrate ion does not engage in any interaction and is disordered over two positions.

The N–H···O hydrogen bonds (Table 1) generate a layer structure; the layers are parallel to [1 0 - 1].

### Experimental

A methanol solution of 2-benzoylpyridine semicarbazone (0.316 g, 1 mmol) and Ni(NO_3_)_2_.6H_2_O (0.290 g, 1 mmol) was heated for 5 h. The brown solid was collected, dried and recrystallized from methanol.

### Refinement

Carbon-bound H-atoms were placed in calculated positions (C–H 0.93 Å) and were included in the refinement in the riding model approximation, with *U*(H) set to 1.2*U*(C).

The amino H-atoms were similarly treated (N–H 0.88 Å) and their temperature factors tied by a factor of 1.2 times.

Omitted owing to bad disagreement were (1 0 1), (-1 0 1), (2 0 0), (0 2 0) and (1 1 1).

Of the two nitrate ions, the one that is engaged in hydrogen bonding is ordered; the other is disordered over two positions in an assumed 1:1 ratio. The N–O bond lengths were restrained to 1.25±0.01 Å and the O···O distances to 2.17±0.01 Å. The temperature factors of the two N atoms were made equal; the anisotropic temperature factors of the disordered atoms were restrained to be nearly isotropic.

The final difference Fourier map had a peak at 1.95 Å from O8.

### Crystal data

**Table:**

[Ni(C_19_H_16_N_4_O)_2_](NO_3_)_2_	*F*(000) = 1688
*M**_r_* = 815.45	*D*_x_ = 1.469 Mg m^−^^3^
Monoclinic, *P*2_1_/*n*	Mo *K*α radiation, λ = 0.71073 Å
Hall symbol: -P 2yn	Cell parameters from 9932 reflections
*a* = 14.0112 (3) Å	θ = 2.2–23.3°
*b* = 16.0445 (3) Å	µ = 0.60 mm^−^^1^
*c* = 16.4939 (3) Å	*T* = 293 K
β = 95.959 (1)°	Prism, brown
*V* = 3687.84 (12) Å^3^	0.35 × 0.30 × 0.20 mm
*Z* = 4	

### Data collection

**Table:**

Bruker APEXII diffractometer	6527 independent reflections
Radiation source: fine-focus sealed tube	4632 reflections with *I* > 2σ(*I*)
Graphite monochromator	*R*_int_ = 0.070
ω scans	θ_max_ = 25.1°, θ_min_ = 2.5°
Absorption correction: multi-scan (*SADABS*; Sheldrick, 1996)	*h* = −16→16
*T*_min_ = 0.819, *T*_max_ = 0.890	*k* = −19→19
51193 measured reflections	*l* = −19→19

### Refinement

**Table:**

Refinement on *F*^2^	Primary atom site location: structure-invariant direct methods
Least-squares matrix: full	Secondary atom site location: difference Fourier map
*R*[*F*^2^ > 2σ(*F*^2^)] = 0.053	Hydrogen site location: inferred from neighbouring sites
*wR*(*F*^2^) = 0.169	H-atom parameters constrained
*S* = 1.09	*w* = 1/[σ^2^(*F*_o_^2^) + (0.0797*P*)^2^ + 3.6302*P*] where *P* = (*F*_o_^2^ + 2*F*_c_^2^)/3
6527 reflections	(Δ/σ)_max_ = 0.001
544 parameters	Δρ_max_ = 1.11 e Å^−^^3^
62 restraints	Δρ_min_ = −0.52 e Å^−^^3^

### Fractional atomic coordinates and isotropic or equivalent isotropic displacement parameters (Å^2^)

**Table:**

	*x*	*y*	*z*	*U*_iso_*/*U*_eq_	Occ. (<1)
Ni1	0.40791 (3)	0.14864 (3)	0.71829 (3)	0.04455 (19)	
O1	0.34631 (18)	0.17104 (18)	0.83225 (16)	0.0502 (7)	
O2	0.42378 (18)	0.28044 (17)	0.70569 (17)	0.0520 (7)	
O3	0.6200 (2)	0.14646 (19)	1.0402 (2)	0.0650 (8)	
O4	0.5145 (2)	0.0619 (2)	1.0767 (3)	0.0911 (12)	
O5	0.6630 (2)	0.0337 (2)	1.1056 (2)	0.0727 (9)	
O6	0.4526 (17)	0.1368 (17)	0.3950 (8)	0.42 (2)	0.50
O7	0.3370 (10)	0.1133 (11)	0.3027 (12)	0.197 (9)	0.50
O8	0.4540 (7)	0.1895 (7)	0.2742 (7)	0.148 (4)	0.50
O6'	0.4334 (8)	0.1360 (6)	0.3839 (5)	0.120 (4)	0.50
O7'	0.2987 (6)	0.1301 (8)	0.3118 (8)	0.133 (5)	0.50
O8'	0.4224 (7)	0.1088 (8)	0.2541 (5)	0.144 (4)	0.50
N1	0.5226 (2)	0.1185 (2)	0.6468 (2)	0.0490 (8)	
N2	0.5168 (2)	0.12888 (18)	0.80258 (18)	0.0422 (7)	
N3	0.4988 (2)	0.13963 (19)	0.88060 (19)	0.0452 (8)	
H3	0.5439	0.1351	0.9217	0.054*	
N4	0.3881 (2)	0.1629 (2)	0.96957 (18)	0.0462 (8)	
H4	0.4375	0.1538	1.0059	0.055*	
N5	0.3430 (2)	0.0325 (2)	0.69143 (18)	0.0456 (7)	
N6	0.2954 (2)	0.18011 (19)	0.64135 (19)	0.0434 (7)	
N7	0.2858 (2)	0.26271 (19)	0.62321 (19)	0.0493 (8)	
H7	0.2390	0.2826	0.5890	0.059*	
N8	0.3438 (2)	0.39382 (19)	0.65125 (19)	0.0476 (8)	
H8	0.2917	0.4096	0.6208	0.057*	
N9	0.5986 (2)	0.0797 (2)	1.0743 (2)	0.0549 (9)	
N10	0.4174 (6)	0.1480 (4)	0.3243 (5)	0.0299 (15)	0.50
N10'	0.3918 (5)	0.1254 (4)	0.3193 (5)	0.0299 (15)	0.50
C1	0.5217 (3)	0.1076 (3)	0.5665 (3)	0.0613 (12)	
H1	0.4648	0.1174	0.5336	0.074*	
C2	0.6004 (4)	0.0827 (3)	0.5304 (3)	0.0708 (13)	
H2	0.5968	0.0749	0.4743	0.085*	
C3	0.6841 (4)	0.0697 (4)	0.5781 (3)	0.0792 (15)	
H3A	0.7393	0.0545	0.5548	0.095*	
C4	0.6863 (3)	0.0793 (3)	0.6617 (3)	0.0631 (12)	
H4A	0.7425	0.0691	0.6954	0.076*	
C5	0.6054 (3)	0.1039 (2)	0.6940 (2)	0.0427 (8)	
C6	0.6015 (3)	0.1138 (2)	0.7824 (2)	0.0418 (8)	
C7	0.6886 (3)	0.1059 (2)	0.8411 (2)	0.0427 (9)	
C8	0.7058 (3)	0.0339 (3)	0.8855 (3)	0.0623 (12)	
H8A	0.6622	−0.0099	0.8795	0.075*	
C9	0.7884 (3)	0.0271 (3)	0.9391 (3)	0.0701 (13)	
H9	0.8007	−0.0220	0.9683	0.084*	
C10	0.8513 (3)	0.0912 (3)	0.9494 (3)	0.0645 (12)	
H10	0.9065	0.0862	0.9856	0.077*	
C11	0.8335 (3)	0.1632 (3)	0.9066 (3)	0.0689 (13)	
H11	0.8760	0.2078	0.9143	0.083*	
C12	0.7525 (3)	0.1700 (3)	0.8520 (3)	0.0605 (11)	
H12	0.7413	0.2189	0.8223	0.073*	
C13	0.4053 (3)	0.1582 (2)	0.8915 (2)	0.0408 (8)	
C14	0.3005 (3)	0.1806 (2)	1.0008 (2)	0.0454 (9)	
C15	0.3018 (4)	0.1819 (3)	1.0851 (3)	0.0622 (11)	
H15	0.3593	0.1745	1.1179	0.075*	
C16	0.2180 (5)	0.1941 (3)	1.1200 (3)	0.0821 (16)	
H16	0.2191	0.1944	1.1764	0.099*	
C17	0.1329 (4)	0.2059 (3)	1.0728 (4)	0.0843 (17)	
H17	0.0764	0.2134	1.0969	0.101*	
C18	0.1316 (3)	0.2065 (3)	0.9904 (4)	0.0747 (14)	
H18	0.0739	0.2155	0.9584	0.090*	
C19	0.2149 (3)	0.1940 (3)	0.9531 (3)	0.0549 (10)	
H19	0.2130	0.1947	0.8965	0.066*	
C20	0.3720 (3)	−0.0421 (3)	0.7173 (3)	0.0581 (11)	
H20	0.4279	−0.0463	0.7526	0.070*	
C21	0.3229 (4)	−0.1138 (3)	0.6941 (3)	0.0682 (13)	
H21	0.3457	−0.1655	0.7127	0.082*	
C22	0.2398 (3)	−0.1075 (3)	0.6430 (3)	0.0655 (12)	
H22	0.2042	−0.1549	0.6278	0.079*	
C23	0.2094 (3)	−0.0307 (2)	0.6144 (3)	0.0527 (10)	
H23	0.1536	−0.0255	0.5790	0.063*	
C24	0.2629 (3)	0.0384 (2)	0.6391 (2)	0.0420 (8)	
C25	0.2392 (3)	0.1235 (2)	0.6092 (2)	0.0430 (9)	
C26	0.1605 (3)	0.1381 (2)	0.5427 (2)	0.0478 (9)	
C27	0.1836 (4)	0.1433 (3)	0.4643 (3)	0.0699 (13)	
H27	0.2476	0.1420	0.4541	0.084*	
C28	0.1123 (4)	0.1506 (3)	0.4003 (3)	0.0824 (16)	
H28	0.1281	0.1538	0.3470	0.099*	
C29	0.0180 (4)	0.1531 (3)	0.4157 (4)	0.0772 (15)	
H29	−0.0301	0.1579	0.3727	0.093*	
C30	−0.0050 (4)	0.1486 (3)	0.4926 (4)	0.0742 (14)	
H30	−0.0691	0.1508	0.5026	0.089*	
C31	0.0658 (3)	0.1408 (3)	0.5571 (3)	0.0662 (12)	
H31	0.0493	0.1373	0.6102	0.079*	
C32	0.3561 (3)	0.3123 (2)	0.6632 (2)	0.0414 (8)	
C33	0.4070 (3)	0.4574 (2)	0.6830 (2)	0.0445 (9)	
C34	0.3701 (3)	0.5357 (3)	0.6889 (3)	0.0646 (12)	
H34	0.3053	0.5453	0.6735	0.077*	
C35	0.4286 (4)	0.6003 (3)	0.7177 (3)	0.0777 (15)	
H35	0.4028	0.6534	0.7219	0.093*	
C36	0.5243 (4)	0.5875 (3)	0.7402 (3)	0.0738 (14)	
H36	0.5638	0.6313	0.7596	0.089*	
C37	0.5600 (4)	0.5102 (4)	0.7338 (4)	0.0853 (17)	
H37	0.6250	0.5011	0.7487	0.102*	
C38	0.5027 (3)	0.4439 (3)	0.7055 (3)	0.0690 (13)	
H38	0.5288	0.3909	0.7019	0.083*	

### Atomic displacement parameters (Å^2^)

**Table:**

	*U*^11^	*U*^22^	*U*^33^	*U*^12^	*U*^13^	*U*^23^
Ni1	0.0350 (3)	0.0502 (3)	0.0475 (3)	−0.0057 (2)	−0.0003 (2)	−0.0044 (2)
O1	0.0369 (14)	0.0726 (18)	0.0388 (14)	−0.0002 (13)	−0.0078 (12)	0.0050 (13)
O2	0.0394 (15)	0.0557 (16)	0.0565 (17)	−0.0020 (12)	−0.0154 (13)	0.0087 (13)
O3	0.066 (2)	0.0626 (19)	0.0613 (19)	−0.0080 (15)	−0.0183 (16)	0.0078 (15)
O4	0.0392 (18)	0.108 (3)	0.121 (3)	−0.0079 (18)	−0.0162 (18)	0.035 (2)
O5	0.0519 (18)	0.081 (2)	0.079 (2)	0.0086 (16)	−0.0194 (16)	0.0208 (17)
O6	0.42 (3)	0.41 (3)	0.42 (3)	0.016 (10)	0.043 (11)	−0.002 (10)
O7	0.207 (12)	0.188 (12)	0.195 (12)	0.014 (9)	0.019 (9)	0.015 (8)
O8	0.137 (7)	0.125 (7)	0.179 (8)	0.041 (6)	−0.002 (7)	−0.009 (7)
O6'	0.144 (7)	0.123 (6)	0.079 (6)	0.042 (5)	−0.044 (5)	0.001 (5)
O7'	0.127 (7)	0.136 (8)	0.139 (8)	0.053 (6)	0.029 (6)	−0.005 (6)
O8'	0.111 (6)	0.193 (8)	0.126 (7)	0.048 (6)	0.008 (6)	0.000 (7)
N1	0.0423 (18)	0.0555 (19)	0.0468 (19)	−0.0035 (15)	−0.0072 (15)	0.0027 (15)
N2	0.0396 (17)	0.0479 (18)	0.0388 (17)	−0.0008 (13)	0.0024 (14)	−0.0015 (13)
N3	0.0348 (16)	0.061 (2)	0.0382 (17)	0.0020 (14)	−0.0044 (13)	−0.0008 (14)
N4	0.0385 (17)	0.067 (2)	0.0322 (17)	0.0053 (15)	−0.0033 (13)	0.0017 (14)
N5	0.0388 (17)	0.056 (2)	0.0399 (17)	−0.0005 (14)	−0.0056 (14)	0.0038 (14)
N6	0.0395 (17)	0.0434 (17)	0.0456 (18)	−0.0035 (14)	−0.0035 (14)	−0.0018 (14)
N7	0.0455 (18)	0.0473 (19)	0.0502 (19)	−0.0050 (14)	−0.0178 (15)	0.0026 (15)
N8	0.0437 (18)	0.0456 (19)	0.0494 (19)	−0.0048 (14)	−0.0144 (14)	0.0063 (15)
N9	0.047 (2)	0.066 (2)	0.048 (2)	−0.0011 (18)	−0.0136 (16)	0.0029 (17)
N10	0.026 (4)	0.025 (3)	0.038 (2)	−0.006 (3)	−0.004 (3)	−0.007 (2)
N10'	0.026 (4)	0.025 (3)	0.038 (2)	−0.006 (3)	−0.004 (3)	−0.007 (2)
C1	0.064 (3)	0.067 (3)	0.049 (3)	−0.011 (2)	−0.014 (2)	0.004 (2)
C2	0.080 (3)	0.091 (4)	0.041 (2)	0.003 (3)	0.006 (2)	−0.005 (2)
C3	0.071 (3)	0.111 (4)	0.058 (3)	0.019 (3)	0.017 (3)	−0.007 (3)
C4	0.053 (2)	0.089 (3)	0.047 (2)	0.022 (2)	0.002 (2)	−0.001 (2)
C5	0.043 (2)	0.046 (2)	0.039 (2)	0.0036 (16)	0.0006 (16)	0.0024 (16)
C6	0.037 (2)	0.042 (2)	0.045 (2)	0.0013 (16)	−0.0004 (16)	0.0036 (16)
C7	0.0356 (19)	0.054 (2)	0.037 (2)	0.0054 (17)	0.0003 (16)	0.0010 (17)
C8	0.051 (2)	0.061 (3)	0.072 (3)	0.000 (2)	−0.008 (2)	0.012 (2)
C9	0.063 (3)	0.077 (3)	0.066 (3)	0.015 (3)	−0.014 (2)	0.019 (2)
C10	0.046 (2)	0.097 (4)	0.048 (2)	0.009 (2)	−0.0087 (19)	0.001 (2)
C11	0.053 (3)	0.081 (3)	0.069 (3)	−0.015 (2)	−0.010 (2)	−0.007 (3)
C12	0.057 (3)	0.062 (3)	0.060 (3)	−0.006 (2)	−0.007 (2)	0.009 (2)
C13	0.0366 (19)	0.043 (2)	0.042 (2)	0.0008 (15)	−0.0016 (16)	−0.0001 (16)
C14	0.049 (2)	0.043 (2)	0.045 (2)	0.0019 (17)	0.0072 (18)	−0.0003 (17)
C15	0.074 (3)	0.066 (3)	0.048 (3)	0.009 (2)	0.010 (2)	0.003 (2)
C16	0.110 (5)	0.082 (4)	0.061 (3)	0.023 (3)	0.038 (3)	0.006 (3)
C17	0.083 (4)	0.077 (3)	0.102 (5)	0.023 (3)	0.050 (4)	0.014 (3)
C18	0.053 (3)	0.078 (3)	0.096 (4)	0.015 (2)	0.022 (3)	0.008 (3)
C19	0.047 (2)	0.056 (2)	0.063 (3)	0.0066 (19)	0.007 (2)	0.007 (2)
C20	0.051 (2)	0.071 (3)	0.051 (2)	0.009 (2)	−0.0049 (19)	0.009 (2)
C21	0.077 (3)	0.053 (3)	0.073 (3)	0.010 (2)	−0.001 (3)	0.010 (2)
C22	0.059 (3)	0.052 (3)	0.084 (3)	−0.005 (2)	−0.001 (2)	−0.004 (2)
C23	0.043 (2)	0.052 (2)	0.061 (3)	−0.0043 (18)	−0.0059 (19)	−0.0068 (19)
C24	0.0369 (19)	0.049 (2)	0.039 (2)	−0.0028 (16)	−0.0016 (16)	−0.0018 (16)
C25	0.038 (2)	0.051 (2)	0.039 (2)	−0.0029 (17)	−0.0029 (16)	−0.0014 (16)
C26	0.046 (2)	0.046 (2)	0.048 (2)	−0.0049 (17)	−0.0147 (18)	−0.0005 (17)
C27	0.059 (3)	0.095 (4)	0.052 (3)	−0.013 (2)	−0.011 (2)	0.006 (2)
C28	0.083 (4)	0.108 (4)	0.051 (3)	−0.018 (3)	−0.018 (3)	0.013 (3)
C29	0.069 (3)	0.077 (3)	0.077 (4)	−0.005 (3)	−0.034 (3)	0.011 (3)
C30	0.047 (3)	0.086 (4)	0.084 (4)	0.000 (2)	−0.020 (2)	0.000 (3)
C31	0.049 (3)	0.084 (3)	0.062 (3)	0.001 (2)	−0.008 (2)	−0.002 (2)
C32	0.037 (2)	0.051 (2)	0.0357 (19)	−0.0021 (17)	−0.0013 (16)	0.0027 (16)
C33	0.043 (2)	0.054 (2)	0.0346 (19)	−0.0107 (17)	−0.0021 (16)	0.0016 (16)
C34	0.062 (3)	0.056 (3)	0.072 (3)	0.000 (2)	−0.016 (2)	0.000 (2)
C35	0.089 (4)	0.061 (3)	0.078 (3)	−0.008 (3)	−0.015 (3)	−0.011 (2)
C36	0.074 (3)	0.075 (3)	0.070 (3)	−0.033 (3)	−0.003 (3)	−0.011 (3)
C37	0.046 (3)	0.086 (4)	0.121 (5)	−0.025 (3)	−0.002 (3)	−0.019 (3)
C38	0.041 (2)	0.070 (3)	0.096 (4)	−0.009 (2)	0.002 (2)	−0.015 (3)

### Geometric parameters (Å, º)

**Table:**

Ni1—N2	1.980 (3)	C9—H9	0.9300
Ni1—N6	1.984 (3)	C10—C11	1.363 (7)
Ni1—N5	2.101 (3)	C10—H10	0.9300
Ni1—O2	2.139 (3)	C11—C12	1.377 (6)
Ni1—N1	2.146 (3)	C11—H11	0.9300
Ni1—O1	2.178 (3)	C12—H12	0.9300
O1—C13	1.229 (4)	C14—C19	1.381 (6)
O2—C32	1.231 (4)	C14—C15	1.389 (6)
O3—N9	1.261 (4)	C15—C16	1.374 (7)
O4—N9	1.217 (4)	C15—H15	0.9300
O5—N9	1.237 (4)	C16—C17	1.367 (8)
O6—N10	1.231 (8)	C16—H16	0.9300
O7—N10	1.274 (8)	C17—C18	1.356 (8)
O8—N10	1.216 (8)	C17—H17	0.9300
O6'—N10'	1.172 (7)	C18—C19	1.390 (6)
O7'—N10'	1.300 (8)	C18—H18	0.9300
O8'—N10'	1.227 (8)	C19—H19	0.9300
N1—C1	1.334 (5)	C20—C21	1.373 (6)
N1—C5	1.348 (5)	C20—H20	0.9300
N2—C6	1.288 (5)	C21—C22	1.369 (7)
N2—N3	1.348 (4)	C21—H21	0.9300
N3—C13	1.374 (5)	C22—C23	1.370 (6)
N3—H3	0.8800	C22—H22	0.9300
N4—C13	1.337 (5)	C23—C24	1.378 (5)
N4—C14	1.408 (5)	C23—H23	0.9300
N4—H4	0.8800	C24—C25	1.478 (5)
N5—C20	1.320 (5)	C25—C26	1.491 (5)
N5—C24	1.346 (5)	C26—C27	1.367 (6)
N6—C25	1.280 (5)	C26—C31	1.373 (6)
N6—N7	1.362 (4)	C27—C28	1.381 (7)
N7—C32	1.379 (5)	C27—H27	0.9300
N7—H7	0.8800	C28—C29	1.371 (8)
N8—C32	1.331 (5)	C28—H28	0.9300
N8—C33	1.415 (5)	C29—C30	1.343 (8)
N8—H8	0.8800	C29—H29	0.9300
C1—C2	1.367 (7)	C30—C31	1.382 (7)
C1—H1	0.9300	C30—H30	0.9300
C2—C3	1.358 (7)	C31—H31	0.9300
C2—H2	0.9300	C33—C34	1.366 (6)
C3—C4	1.385 (6)	C33—C38	1.371 (6)
C3—H3A	0.9300	C34—C35	1.375 (6)
C4—C5	1.360 (5)	C34—H34	0.9300
C4—H4A	0.9300	C35—C36	1.369 (7)
C5—C6	1.474 (5)	C35—H35	0.9300
C6—C7	1.483 (5)	C36—C37	1.345 (7)
C7—C12	1.363 (6)	C36—H36	0.9300
C7—C8	1.375 (6)	C37—C38	1.384 (6)
C8—C9	1.387 (6)	C37—H37	0.9300
C8—H8A	0.9300	C38—H38	0.9300
C9—C10	1.354 (7)		
			
N2—Ni1—N6	173.28 (12)	C12—C11—H11	120.0
N2—Ni1—N5	106.93 (12)	C7—C12—C11	120.9 (4)
N6—Ni1—N5	78.05 (12)	C7—C12—H12	119.6
N2—Ni1—O2	98.41 (11)	C11—C12—H12	119.6
N6—Ni1—O2	76.70 (11)	O1—C13—N4	125.6 (3)
N5—Ni1—O2	154.66 (11)	O1—C13—N3	120.3 (3)
N2—Ni1—N1	77.50 (12)	N4—C13—N3	114.1 (3)
N6—Ni1—N1	107.25 (12)	C19—C14—C15	119.3 (4)
N5—Ni1—N1	90.96 (12)	C19—C14—N4	124.1 (4)
O2—Ni1—N1	94.48 (12)	C15—C14—N4	116.6 (4)
N2—Ni1—O1	76.45 (11)	C16—C15—C14	119.8 (5)
N6—Ni1—O1	98.65 (11)	C16—C15—H15	120.1
N5—Ni1—O1	97.27 (11)	C14—C15—H15	120.1
O2—Ni1—O1	88.58 (11)	C17—C16—C15	120.9 (5)
N1—Ni1—O1	153.94 (11)	C17—C16—H16	119.5
C13—O1—Ni1	111.4 (2)	C15—C16—H16	119.5
C32—O2—Ni1	112.6 (2)	C18—C17—C16	119.4 (5)
C1—N1—C5	118.0 (4)	C18—C17—H17	120.3
C1—N1—Ni1	130.1 (3)	C16—C17—H17	120.3
C5—N1—Ni1	111.7 (2)	C17—C18—C19	121.2 (5)
C6—N2—N3	122.6 (3)	C17—C18—H18	119.4
C6—N2—Ni1	120.8 (3)	C19—C18—H18	119.4
N3—N2—Ni1	116.3 (2)	C14—C19—C18	119.3 (4)
N2—N3—C13	115.3 (3)	C14—C19—H19	120.4
N2—N3—H3	122.4	C18—C19—H19	120.4
C13—N3—H3	122.4	N5—C20—C21	122.8 (4)
C13—N4—C14	127.9 (3)	N5—C20—H20	118.6
C13—N4—H4	116.0	C21—C20—H20	118.6
C14—N4—H4	116.0	C22—C21—C20	118.6 (4)
C20—N5—C24	118.7 (3)	C22—C21—H21	120.7
C20—N5—Ni1	128.6 (3)	C20—C21—H21	120.7
C24—N5—Ni1	112.7 (2)	C21—C22—C23	119.5 (4)
C25—N6—N7	123.8 (3)	C21—C22—H22	120.2
C25—N6—Ni1	119.9 (3)	C23—C22—H22	120.2
N7—N6—Ni1	116.2 (2)	C22—C23—C24	118.8 (4)
N6—N7—C32	114.0 (3)	C22—C23—H23	120.6
N6—N7—H7	123.0	C24—C23—H23	120.6
C32—N7—H7	123.0	N5—C24—C23	121.6 (4)
C32—N8—C33	126.0 (3)	N5—C24—C25	115.1 (3)
C32—N8—H8	117.0	C23—C24—C25	123.3 (3)
C33—N8—H8	117.0	N6—C25—C24	114.2 (3)
O4—N9—O5	120.9 (4)	N6—C25—C26	124.8 (3)
O4—N9—O3	119.4 (4)	C24—C25—C26	120.9 (3)
O5—N9—O3	119.7 (4)	C27—C26—C31	119.4 (4)
O8—N10—O6	124.6 (10)	C27—C26—C25	118.4 (4)
O8—N10—O7	118.2 (8)	C31—C26—C25	122.1 (4)
O6—N10—O7	117.2 (9)	C26—C27—C28	120.3 (5)
O6'—N10'—O8'	130.0 (8)	C26—C27—H27	119.9
O6'—N10'—O7'	118.5 (8)	C28—C27—H27	119.9
O8'—N10'—O7'	111.6 (7)	C29—C28—C27	119.7 (5)
N1—C1—C2	123.1 (4)	C29—C28—H28	120.2
N1—C1—H1	118.5	C27—C28—H28	120.2
C2—C1—H1	118.5	C30—C29—C28	120.2 (5)
C3—C2—C1	118.7 (4)	C30—C29—H29	119.9
C3—C2—H2	120.7	C28—C29—H29	119.9
C1—C2—H2	120.7	C29—C30—C31	120.5 (5)
C2—C3—C4	119.2 (4)	C29—C30—H30	119.7
C2—C3—H3A	120.4	C31—C30—H30	119.7
C4—C3—H3A	120.4	C26—C31—C30	119.9 (5)
C5—C4—C3	119.3 (4)	C26—C31—H31	120.1
C5—C4—H4A	120.4	C30—C31—H31	120.1
C3—C4—H4A	120.4	O2—C32—N8	124.9 (3)
N1—C5—C4	121.7 (4)	O2—C32—N7	120.1 (3)
N1—C5—C6	115.8 (3)	N8—C32—N7	115.0 (3)
C4—C5—C6	122.5 (3)	C34—C33—C38	119.5 (4)
N2—C6—C5	114.0 (3)	C34—C33—N8	117.6 (4)
N2—C6—C7	124.4 (3)	C38—C33—N8	122.9 (4)
C5—C6—C7	121.6 (3)	C33—C34—C35	120.1 (4)
C12—C7—C8	119.1 (4)	C33—C34—H34	120.0
C12—C7—C6	120.5 (4)	C35—C34—H34	120.0
C8—C7—C6	120.4 (4)	C36—C35—C34	120.8 (5)
C7—C8—C9	119.6 (4)	C36—C35—H35	119.6
C7—C8—H8A	120.2	C34—C35—H35	119.6
C9—C8—H8A	120.2	C37—C36—C35	118.6 (4)
C10—C9—C8	120.6 (4)	C37—C36—H36	120.7
C10—C9—H9	119.7	C35—C36—H36	120.7
C8—C9—H9	119.7	C36—C37—C38	121.8 (5)
C9—C10—C11	119.8 (4)	C36—C37—H37	119.1
C9—C10—H10	120.1	C38—C37—H37	119.1
C11—C10—H10	120.1	C33—C38—C37	119.1 (5)
C10—C11—C12	119.9 (4)	C33—C38—H38	120.4
C10—C11—H11	120.0	C37—C38—H38	120.4
			
N2—Ni1—O1—C13	−2.4 (2)	C5—C6—C7—C8	−101.7 (5)
N6—Ni1—O1—C13	−177.7 (3)	C12—C7—C8—C9	−1.3 (7)
N5—Ni1—O1—C13	103.3 (3)	C6—C7—C8—C9	179.0 (4)
O2—Ni1—O1—C13	−101.4 (3)	C7—C8—C9—C10	1.3 (7)
N1—Ni1—O1—C13	−4.1 (4)	C8—C9—C10—C11	−0.1 (8)
N2—Ni1—O2—C32	−171.1 (3)	C9—C10—C11—C12	−1.2 (8)
N6—Ni1—O2—C32	4.2 (3)	C8—C7—C12—C11	0.1 (7)
N5—Ni1—O2—C32	9.0 (4)	C6—C7—C12—C11	179.8 (4)
N1—Ni1—O2—C32	110.9 (3)	C10—C11—C12—C7	1.1 (7)
O1—Ni1—O2—C32	−95.1 (3)	Ni1—O1—C13—N4	−176.8 (3)
N2—Ni1—N1—C1	174.4 (4)	Ni1—O1—C13—N3	5.3 (4)
N6—Ni1—N1—C1	−10.5 (4)	C14—N4—C13—O1	2.0 (6)
N5—Ni1—N1—C1	67.3 (4)	C14—N4—C13—N3	180.0 (3)
O2—Ni1—N1—C1	−88.0 (4)	N2—N3—C13—O1	−6.3 (5)
O1—Ni1—N1—C1	176.1 (3)	N2—N3—C13—N4	175.6 (3)
N2—Ni1—N1—C5	−1.0 (2)	C13—N4—C14—C19	2.0 (6)
N6—Ni1—N1—C5	174.1 (2)	C13—N4—C14—C15	−179.9 (4)
N5—Ni1—N1—C5	−108.1 (3)	C19—C14—C15—C16	1.8 (7)
O2—Ni1—N1—C5	96.6 (3)	N4—C14—C15—C16	−176.4 (4)
O1—Ni1—N1—C5	0.7 (4)	C14—C15—C16—C17	−0.6 (8)
N5—Ni1—N2—C6	91.4 (3)	C15—C16—C17—C18	−0.9 (8)
O2—Ni1—N2—C6	−88.5 (3)	C16—C17—C18—C19	1.1 (8)
N1—Ni1—N2—C6	4.3 (3)	C15—C14—C19—C18	−1.5 (6)
O1—Ni1—N2—C6	−175.0 (3)	N4—C14—C19—C18	176.5 (4)
N5—Ni1—N2—N3	−94.4 (3)	C17—C18—C19—C14	0.1 (7)
O2—Ni1—N2—N3	85.7 (2)	C24—N5—C20—C21	−1.2 (6)
N1—Ni1—N2—N3	178.4 (3)	Ni1—N5—C20—C21	−179.2 (3)
O1—Ni1—N2—N3	−0.8 (2)	N5—C20—C21—C22	−1.0 (7)
C6—N2—N3—C13	177.8 (3)	C20—C21—C22—C23	2.0 (7)
Ni1—N2—N3—C13	3.7 (4)	C21—C22—C23—C24	−1.0 (7)
N2—Ni1—N5—C20	−6.7 (4)	C20—N5—C24—C23	2.3 (6)
N6—Ni1—N5—C20	178.0 (4)	Ni1—N5—C24—C23	−179.4 (3)
O2—Ni1—N5—C20	173.1 (3)	C20—N5—C24—C25	−176.2 (3)
N1—Ni1—N5—C20	70.5 (4)	Ni1—N5—C24—C25	2.1 (4)
O1—Ni1—N5—C20	−84.7 (3)	C22—C23—C24—N5	−1.2 (6)
N2—Ni1—N5—C24	175.2 (2)	C22—C23—C24—C25	177.2 (4)
N6—Ni1—N5—C24	−0.1 (2)	N7—N6—C25—C24	−179.3 (3)
O2—Ni1—N5—C24	−5.0 (4)	Ni1—N6—C25—C24	3.9 (4)
N1—Ni1—N5—C24	−107.6 (3)	N7—N6—C25—C26	5.4 (6)
O1—Ni1—N5—C24	97.2 (3)	Ni1—N6—C25—C26	−171.4 (3)
N5—Ni1—N6—C25	−2.2 (3)	N5—C24—C25—N6	−3.9 (5)
O2—Ni1—N6—C25	175.7 (3)	C23—C24—C25—N6	177.6 (4)
N1—Ni1—N6—C25	85.1 (3)	N5—C24—C25—C26	171.7 (3)
O1—Ni1—N6—C25	−97.9 (3)	C23—C24—C25—C26	−6.8 (6)
N5—Ni1—N6—N7	−179.3 (3)	N6—C25—C26—C27	80.5 (5)
O2—Ni1—N6—N7	−1.4 (2)	C24—C25—C26—C27	−94.6 (5)
N1—Ni1—N6—N7	−92.0 (3)	N6—C25—C26—C31	−103.9 (5)
O1—Ni1—N6—N7	85.1 (3)	C24—C25—C26—C31	81.0 (5)
C25—N6—N7—C32	−178.2 (3)	C31—C26—C27—C28	−0.5 (7)
Ni1—N6—N7—C32	−1.3 (4)	C25—C26—C27—C28	175.2 (4)
C5—N1—C1—C2	−0.2 (6)	C26—C27—C28—C29	0.4 (8)
Ni1—N1—C1—C2	−175.3 (4)	C27—C28—C29—C30	0.1 (8)
N1—C1—C2—C3	−1.1 (8)	C28—C29—C30—C31	−0.5 (8)
C1—C2—C3—C4	2.1 (8)	C27—C26—C31—C30	0.1 (7)
C2—C3—C4—C5	−1.9 (8)	C25—C26—C31—C30	−175.5 (4)
C1—N1—C5—C4	0.4 (6)	C29—C30—C31—C26	0.4 (7)
Ni1—N1—C5—C4	176.4 (3)	Ni1—O2—C32—N8	174.0 (3)
C1—N1—C5—C6	−177.8 (3)	Ni1—O2—C32—N7	−6.4 (4)
Ni1—N1—C5—C6	−1.8 (4)	C33—N8—C32—O2	1.9 (6)
C3—C4—C5—N1	0.6 (7)	C33—N8—C32—N7	−177.8 (3)
C3—C4—C5—C6	178.7 (4)	N6—N7—C32—O2	5.4 (5)
N3—N2—C6—C5	179.8 (3)	N6—N7—C32—N8	−175.0 (3)
Ni1—N2—C6—C5	−6.4 (4)	C32—N8—C33—C34	−158.0 (4)
N3—N2—C6—C7	0.4 (6)	C32—N8—C33—C38	23.7 (6)
Ni1—N2—C6—C7	174.2 (3)	C38—C33—C34—C35	−0.3 (7)
N1—C5—C6—N2	5.2 (5)	N8—C33—C34—C35	−178.6 (4)
C4—C5—C6—N2	−173.0 (4)	C33—C34—C35—C36	0.4 (8)
N1—C5—C6—C7	−175.4 (3)	C34—C35—C36—C37	−0.1 (8)
C4—C5—C6—C7	6.4 (6)	C35—C36—C37—C38	−0.3 (9)
N2—C6—C7—C12	−102.0 (5)	C34—C33—C38—C37	−0.2 (7)
C5—C6—C7—C12	78.6 (5)	N8—C33—C38—C37	178.1 (4)
N2—C6—C7—C8	77.7 (5)	C36—C37—C38—C33	0.5 (9)

### Hydrogen-bond geometry (Å, º)

**Table:**

*D*—H···*A*	*D*—H	H···*A*	*D*···*A*	*D*—H···*A*
N3—H3···O3	0.88	2.14	2.983 (4)	162
N4—H4···O4	0.88	2.11	2.869 (5)	145
N7—H7···O3^i^	0.88	2.11	2.955 (4)	162
N8—H8···O5^i^	0.88	2.01	2.819 (4)	152

Symmetry code: (i) *x*−1/2, −*y*+1/2, *z*−1/2.
